# A high precision method for length-based separation of carbon nanotubes using bio-conjugation, SDS-PAGE and silver staining

**DOI:** 10.1371/journal.pone.0197972

**Published:** 2018-06-25

**Authors:** Zahra Borzooeian, Mohammad E. Taslim, Omid Ghasemi, Saina Rezvani, Giti Borzooeian, Amirhasan Nourbakhsh

**Affiliations:** 1 Department of Mechanical and Industrial Engineering, College of Engineering, Northeastern University, Boston, MA, United States of America; 2 Merrimack Pharmaceuticals Inc, Cambridge, MA, United States of America; 3 Department of Computer Science, Worcester Polytechnic Institute, Worcester, MA, United States of America; 4 Department of Biology, Payamnoor, University of Esfahan, Esfahan, Iran; 5 Department of Electrical Engineering Computer Science, Massachusetts Institute of Technology, Boston, MA, United States of America; Universidad de Castilla-La Mancha, SPAIN

## Abstract

Parametric separation of carbon nanotubes, especially based on their length is a challenge for a number of nano-tech researchers. We demonstrate a method to combine bio-conjugation, SDS-PAGE, and silver staining in order to separate carbon nanotubes on the basis of length. Egg-white lysozyme, conjugated covalently onto the single-walled carbon nanotubes surfaces using carbodiimide method. The proposed conjugation of a biomolecule onto the carbon nanotubes surfaces is a novel idea and a significant step forward for creating an indicator for length-based carbon nanotubes separation. The conjugation step was followed by SDS-PAGE and the nanotube fragments were precisely visualized using silver staining. This high precision, inexpensive, rapid and simple separation method obviates the need for centrifugation, additional chemical analyses, and expensive spectroscopic techniques such as Raman spectroscopy to visualize carbon nanotube bands. In this method, we measured the length of nanotubes using different image analysis techniques which is based on a simplified hydrodynamic model. The method has high precision and resolution and is effective in separating the nanotubes by length which would be a valuable quality control tool for the manufacture of carbon nanotubes of specific lengths in bulk quantities. To this end, we were also able to measure the carbon nanotubes of different length, produced from different sonication time intervals.

## Introduction

Various techniques of synthesizing carbon nanotubes (CNTs) produce nanotubes with different lengths, diameters, and structures. In all applications, when nanotubes are used (conductive and high-strength composites, nanometer-sized semiconductor devices, probes and interconnects [1], sensors [2], energy storage and energy conversion devices, hydrogen storage [3–5], nanotube transistors [6, 7], and nanomedicine [8] structural parameters have considerable impact on the reactivity of carbon nanotubes.

Any new technique that is used to separate and purify nanotubes in a scalable, reproducible, and simple manner needs to measure the morphological parameters (diameter and length) of CNTs. Length of nanotubes have been demonstrated to have positive correlation on thermal and electrical activities, with longer multi-wall carbon nanotubes (MWCNTs) resulting in higher thermal and electrical conductivities [9]. In addition, length of nanotubes impacts transistors performance [10] electromagnetic interference shielding and mechanical properties of CNT-based epoxy composites [11]. Length of nanotubes can also have a significant effect on biological response and human and environmental health. There is a growing consensus that characterization is an essential part of assessing the potential toxicity of nanomaterials in biological systems [12]. Cheng [13] in a study on the influence of carbon nanotube length on toxicity of zebra fish embryos showed that length plays an important role in the in vivo toxicity of functionalized CNTs. One research on single-walled carbon nanotubes (SWCNTs) cytotoxicity showed that the cytotoxic response of cells in a culture is dependent on the degree of functionalization [14] which in turn depends on the length of CNTs. Therefore, length measurement is needed to control and reduce the toxicity to achieve green chemistry of CNTs.

In light of such facts, length-based separation as well as length measurement of CNTs after synthesis has attracted particular attention. However, measuring basic parameters, especially diameter and length of CNTs remains an ongoing challenge to the nanotechnology researchers. Some purification and size-selection techniques consist of solvent (CS2, toluene) treatment of raw nanotubes followed by ultrafiltration [15, 16], floculation using aqueous surfactants [17], oxidation and acid washing coupled with centrifugation, resuspension in surfactant solution, and cross-flow filtration steps [18, 19], polymer suspensions [20, 21], chromatographic purification [22, 23], field-flow fractionation on purified shortened nanotubes [24], and size-exclusion chromatography on raw nanotubes suspended in sodium dodecyl sulfate (SDS) [25, 26].

Electrophoretic methods are known to enable nanomaterials purification [27–29] and characterization [30, 31] as well as their separation based on size, shape, length, and diameter [27, 31]. For diameter-, length-, and curvature-based separation of CNTS, electrophoresis techniques such as AC electrophoresis in isopropyl alcohol to purify MWCNTs [29], capillary electrophoresis to separate SDS-coated SWNCTs [27], and agarose gel electrophoresis to fractionate SWCNT/nucleic acid complexes [32] have been demonstrated. Capillary electrophoresis has also been employed to detect the complexity of DNA-suspended SWCNTs via streptavidin/biotin binding [33]. Despite all the reported works, to the best of authors’ knowledge, there exit no preparative electrophoretic methods for precise and rapid length-based separation of CNTs.

Here, we conjugated lysozyme onto SWCNTs covalently and used sodium dodecyl sulphate polyacrylamide gel electrophoresis (SDS-PAGE) and silver staining to length-based separate conjugated lysozyme-SWCNTs. Lysozyme was attached onto the CNTs as an indicator and horn sonication was used to create CNTs with different lengths. SDS-PAGE separation of bio-conjugated SWCNTs was used according to the charge- and size-dependent mobility of bio-conjugated CNTs under the influence of an applied electric field. The proposed method i.e. conjugation of a biomolecule onto the CNTs surface is a novel idea and a significant step forward in creating an indicator for length-based CNT separation in an electrical field. We used silver staining as a precise staining method to visualize separated CNTs with different lengths. Our results showed that SDS-PAGE with rapid-resolution separation capability and high resolution silver staining for visualization can be used efficiently and effectively to separate CNTs into discrete fractions of uniform length. In addition to ImageJ, a programming code (developed in MATLAB) were used for measuring the intensity signal of dye distribution. Consequently, the concentration of SWCNTs was computed in every band of gel. SWCNTs lengths were determined using a simplified hydrodynamic model [34].

## Materials and methods

Lyophilized chicken egg white lysozyme (EC 3.2.1.17) was purchased from Inovatech, Inc. (Abbotsford, BC, Canada), and Micrococcus lysodeikticus cells, obtained from Sigma-Aldrich Corporation (St. Louis, MO) as salt-free and dry powder, were used without further purification. Carboxyl single-walled carbon nanotubes (SWCNT-COOH) with outer diameter of 1–2 nm were purchased from MKnano, Canada. MES [2-(N-morpholino) ethane sulfonic acid] buffer, N-ethyl-N’-(3-(dimethyl amino) propyl) carbodiimide hydrochloride (EDC), Tris-hydroxymethyl aminomethane (Tris), N,N methylenebisacrylamide (Bis), acrylamide, sodium dodecyl sulfate, ammonium persulfate, tetramethylethylenediamine (TEMED), 2-mercaptoethanol(2ME), 3,3–5,5 tetrabromophenolsulfonphthalein (Bromophenol Blue) and all other chemicals were purchased from Sigma-Aldrich Corporation and used as received.

### Enzyme attachment onto SWCNTs

Details of chemically bonding lysozyme to SWCNTs were given in our previous work [35]. Lysozyme conjugation onto SWCNTs was achieved using carbodiimide method [36]. The activated SWCNTs were dispersed in MES buffer, 50 mM, pH 6.2 (1 mg/mL) and added to an equal volume of 400 mM N-hydroxysuccinimide (NHS) in MES buffer. For coupling of NHS to the carboxylic groups on the surface of nanotubes, 20 mM N-ethyl-N’- (3-(dimethylamino) propyl) carbodiimide hydrochloride (EDC) was added to the mixture. The resulting mixture was stirred at 200 rpm for 30 min followed by sonication (MSE Ultrasonic Disintegrators, 150 W, England) for around 30 min. The resulting mixture was centrifuged at 7000 rpm for 15 min. The centrifuge steps were repeated three times to remove excess EDC and NHS. The enzyme solution (10 mg/ml, 10mM phosphate buffer, pH 8) was added to the rinsed nanotubes and sonicated for ca. 1 min to redisperse the SWCNTs. The mixture was shaken in an orbital shaker at 200 rpm at room temperature during the conjugation process. The conjugated lysozyme-SWCNTs solution was centrifuged and then washed three times with triply distilled water and once with 1% (v/v) Tween-20 to completely remove all nonspecifically adsorbed enzyme. Control enzyme-nanotube conjugates were prepared using the same procedure, only without using EDC and NHS.

### Conjugated lysozyme-SWCNTs characterization

The morphology of conjugated lysozyme-SWCNT was compared with that of activated SWCNTs using time scanning electron microscopy (SEM, S360 Oxford), X-ray diffraction (XRD, D8, Advance, Bruker, axs) at λ = 0.1542 nm, and FTIR spectroscopy (Shimadzu FTIR 8300 spectrophotometer) were employed for characterizing conjugated lysozyme-SWCNTs. Conjugated lysozyme-SWCNT samples were sonicated for three time periods of 3, 7 and 10 minutes.

### SDS-PAGE electrophoresis and silver staining

Acrylamide (29.2 g) and Bis (0.8g) were dissolved in 100 ml water and then filtered to prepare gel stock solution (30%, m/v). The separating gel solution was prepared by mixing 10.0 ml gel stock solution, 10.0 ml Tris–HCl (1.5 mol L^−1^, pH 8.80), 200–800 μl (NH_4_)_2_S_2_O_8_ (10% m /v) and 0.4g SDS and then diluting with water to 40 mL. To prepare stacking gel, 1.33 ml of the gel stock solution was mixed with 2.5 ml Tris–HCl (0.5 mol L^−1^, pH 6.80) and 50 μL (NH_4_)_2_S_2_O_8_ (10%, m/v), and then diluted with water to 10.0 mL. Finally, 10 μL TEMED was added to the mixtures. Before electrophoresis, samples were washed several times with phosphate buffer (10 mM, pH 8) to remove any physically adsorbed enzyme. The electrophoresis buffer was prepared by dissolving Tris (15.14g), glycine (72.05g), and SDS (5g) in 500 ml distilled water. Solution’s pH was adjusted to 8.30. A vertical polyacrylamide gel system was used, consisting of separating (10.0% m/v) and stacking (3.0%, m/v) gels. The sample loading volume was 15 μL. The gels were stained with Coomassie Brilliant Blue R-250. Silver staining of gels was achieved through the Blum method [37]. The procedure consists of fixing with methanol, acetic acid and paraformaldehyde solutions, washing with ethanol (50% and 30%) and ddH_2_O), sensitizing with Na_2_S_2_O_3_.5H_2_O, washing with ddH_2_O, impregnating with silver nitrate and paraformaldehyde solution, washing with ddH_2_O, developing with Na_2_CO_3_, paraformaldehyde and Na_2_S_2_O_3_.5H_2_O solution, washing with ddH_2_O, and ending reaction with a stop solution- methanol 50%, and acetic acid 12%.

### Length measurement using image analysis techniques

Two methods were used to analyze the data of gels, a manual approach using ImageJ and semi-automated method using MATLAB. ImageJ is an FDA & NIH approved software for the analysis of different types of images used in biological sciences such as Western Blot, Digital Pathology, etc. In this study, ImageJ is used to extract two types of information from electrophoresis gels: the distance that each band is moved from the center of CNTs well and the intensity of the signal at each band of the columns. After the calibration for the size, the image is transformed into an 8bit image and the intensities inverted. Then on each lane, a narrow rectangular box is selected along the width of the gel, parallel to the movement on CNTs in the lane and the profile of the intensities are extracted from that box. Using the distances of each band of the lane from the center of the well and the electrical voltages applied to the gel, mobility and consequently length of the CNTs were calculated using Usrey’s equation [34]. In order to extract the intensities of all pixels at each band instead of the rectangular box, a MATLAB code was developed to measure the intensities and the distances automatically. The input of this code was the 8 bit inverted image and the output was the distance of each band from the center of the well and the average intensity of the pixels of each band.

## Results

**Fig 1:**
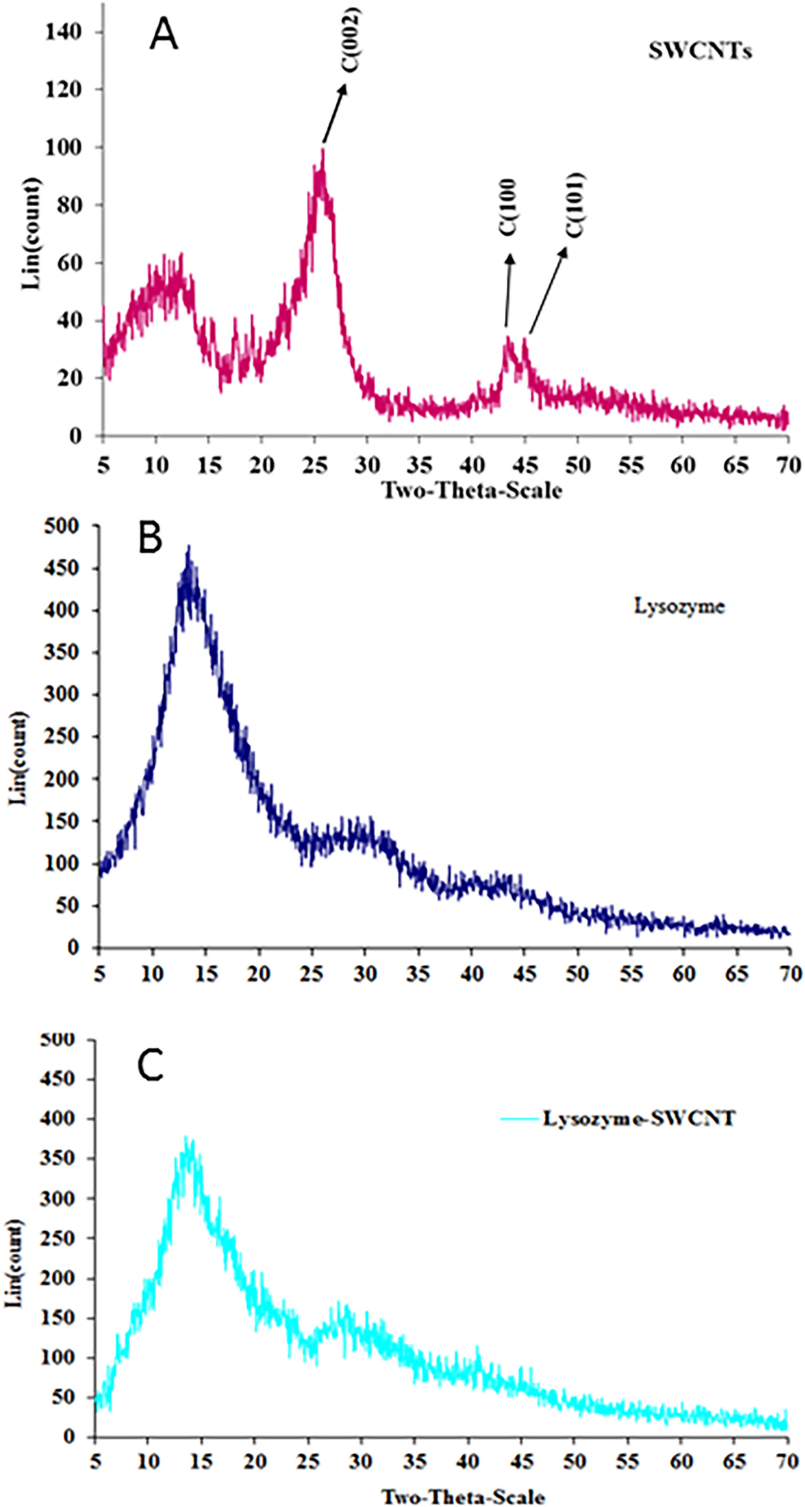
X-ray diffraction (XRD) patterns of A) SWCNTs, B) lysozyme, and C) Conjugated lysozyme-SWCNTs.

Bio-conjugation of lysozyme onto nanotubes surface was achieved using carbodiimide. The interactions between free lysozyme and lysozyme-SWCNTs were analysed using SEM micrographs, XRD and FTIR. XRD pattern, as shown in Fig 1, confirmed the enzyme attachment onto the surface of activated SWCNTs. The strong peaks of the SWCNTs correspond to the (002), (100) and (101) carbon planes. For conjugated lysozyme, the characteristic peaks occur at 2θ of, 14.0, 30.0 and 42.0 which are the same as those for free lysozyme XRD pattern. Indeed, the results showed that there is no significant difference in XRD patterns between the free and conjugated lysozymes, revealing either the adsorption or absorption of lysozyme onto SWCNTs. These results indicate that the conjugation of lysozyme with SWCNTs does not cause a phase change in lysozyme. Therefore, it seems that lysozyme did not have any structural denaturation and could preserve its activity during the conjugation.

**Fig 2:**
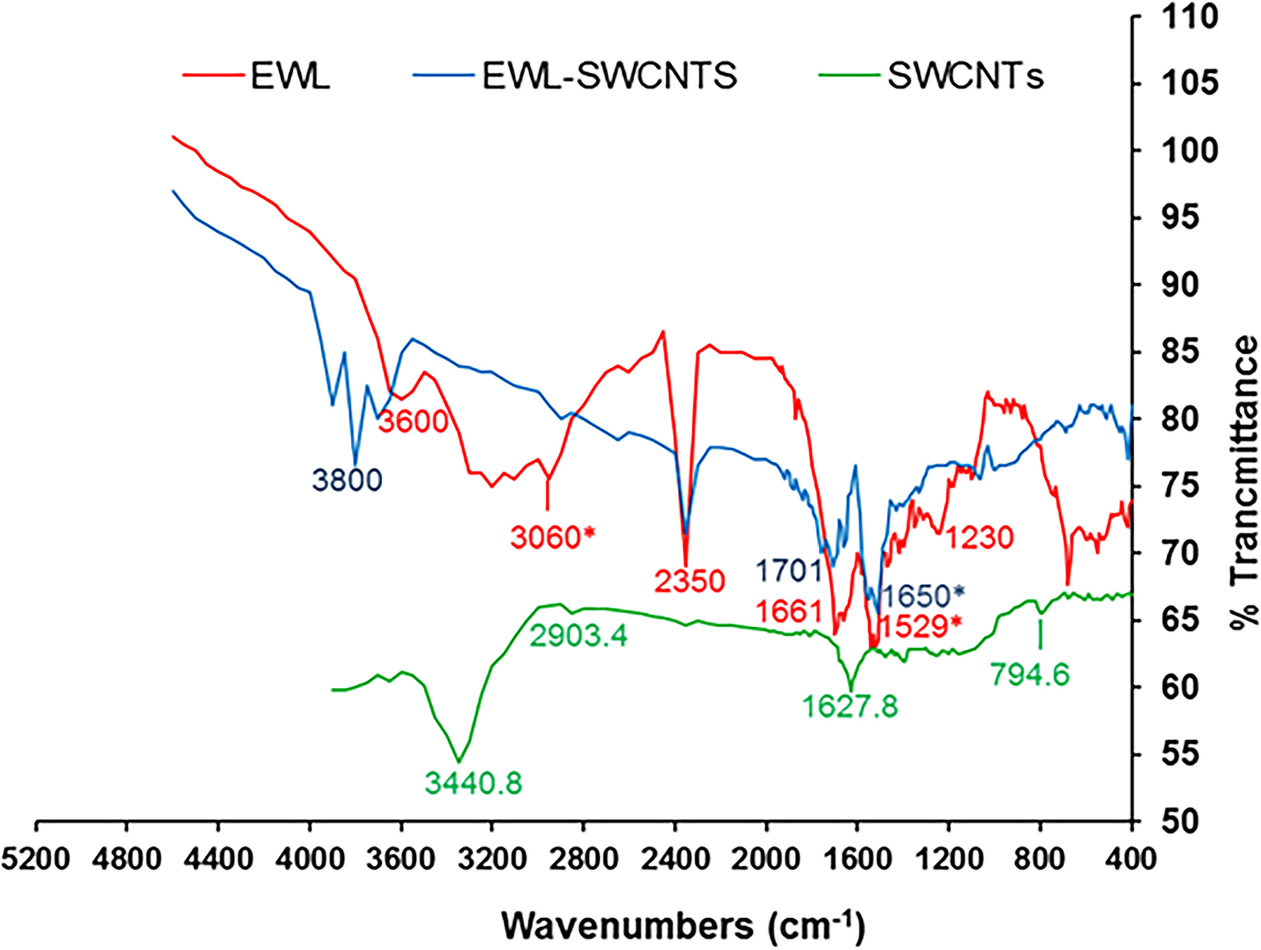
FTIR spectra showing amide bonds in the conjugated lysozyme-SWCNTs. FTIR spectrum for free lysozyme (red), the SWCNTs (green) and conjugated lysozyme-SWCNT blue).

We also studied the mechanism of conjugation of lysozyme to SWCNTs by FTIR analyses. The amide linkages between amino acid residues in polypeptides and proteins result in FTIR fingerprint [38]. The positions of the amide type I and II bands in the FTIR spectra of proteins are indicators of the conformational changes in the protein secondary structure[39] and have been used in studies to investigate immobilized enzyme molecules. FTIR spectra for free lysozyme (red), the SWCNTs (green) and conjugated lysozyme-SWCNT (blue) are shown in Fig 2. Activation of SWCNTs was evidenced via formation of–COOH functional groups in the SWCNT matrix according to the absorption peaks, positioned at 1627.8 cm^-1^ and 3440.8 cm^-1^ (green curve). In lysozyme spectrum, a broad and strong NH_3_ stretching band in the 2950–2600 cm^-1^ region is the characteristic of amino acids. Overtone region expands the absorption to about 2000 cm^-1^. These overtone bands usually contain a prominent band near 2222–2000 cm^-1^ assigned to a combination of asymmetrical NH_3_^+^ bending vibration and the torsional oscillation of the NH_3_^+^ group[40]. Besides, a weak asymmetric NH_3_^+^ bending band is observed near 1661 cm^-1^ and a fairly strong symmetric bending band near 1529 cm^-1^. A peak at 3600 cm^-1^ is a representative of the stretching of N-H group and a peak at 1230 cm^-1^ is a representative of the stretching of C-N group in amine groups. In lysozyme-SWCNTs spectrum, the loss of these peaks is an indication of amide bonds formation between amine groups of lysozyme and carboxyl groups of activated SWCNTs. Also, the absorption peak at 1650 cm^-1^ is a sign of the stretching vibration mode of C = O and peaks at 3800 and 1650 cm^-1^ are represents of the stretching of the N-H groups in the amide group. In conclusion, FTIR spectrum of lysozyme-SWCNTs, confirmed amide bonds formation between amine groups of lysozyme and carboxyl groups of activated SWCNTs.

**Fig 3:**
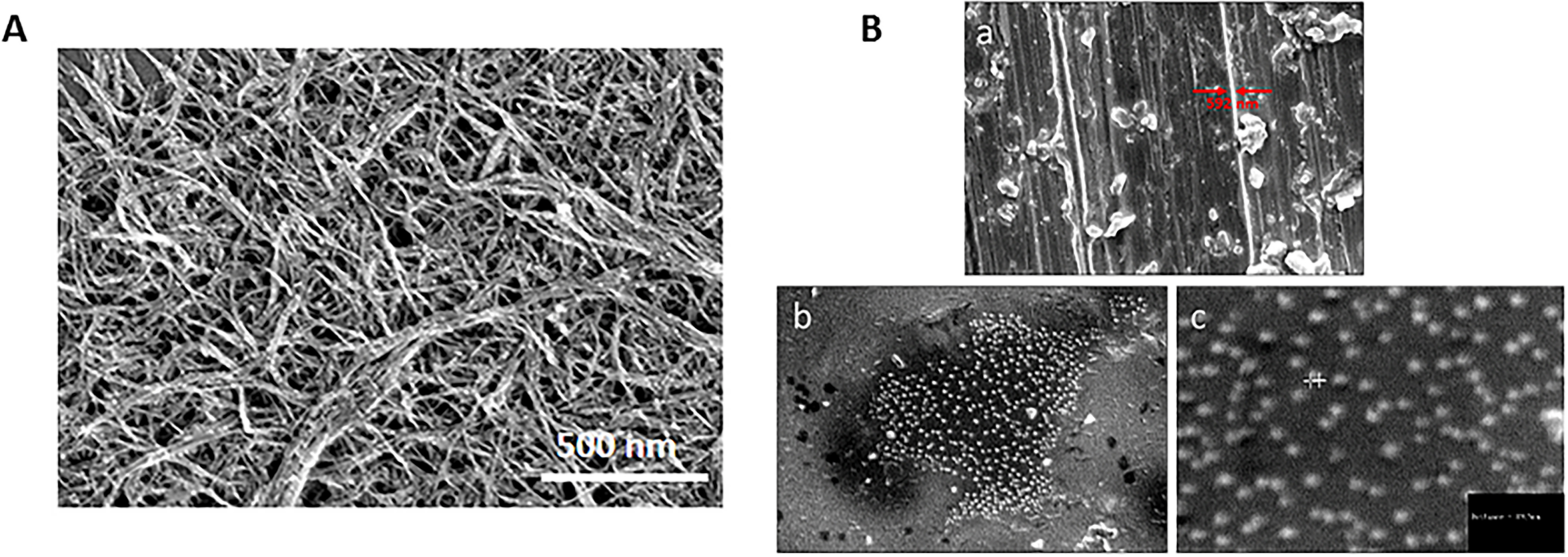
A) SEM image of SWCNTs before conjugation B) SEM image of conjugated lysozyme-SWCNTs at a) a magnification 1.50K, Diameter of the SWCNT bundle ≈ 592 nm, b) a magnification of 10 K, c) a magnification 30.0K, diameter of conjugated lysozyme-SWCNT ≈ 89.5 nm.

Fig 3A, 3B, and 3C show SEM micrographs indicating the size and morphology of the conjugated lysozyme-SWCNTs. Increased sidewall thickness of the conjugated lysozyme-SWCNTs to about 89.5–95 nm is an indication of a successful conjugation.

**Fig 4:**
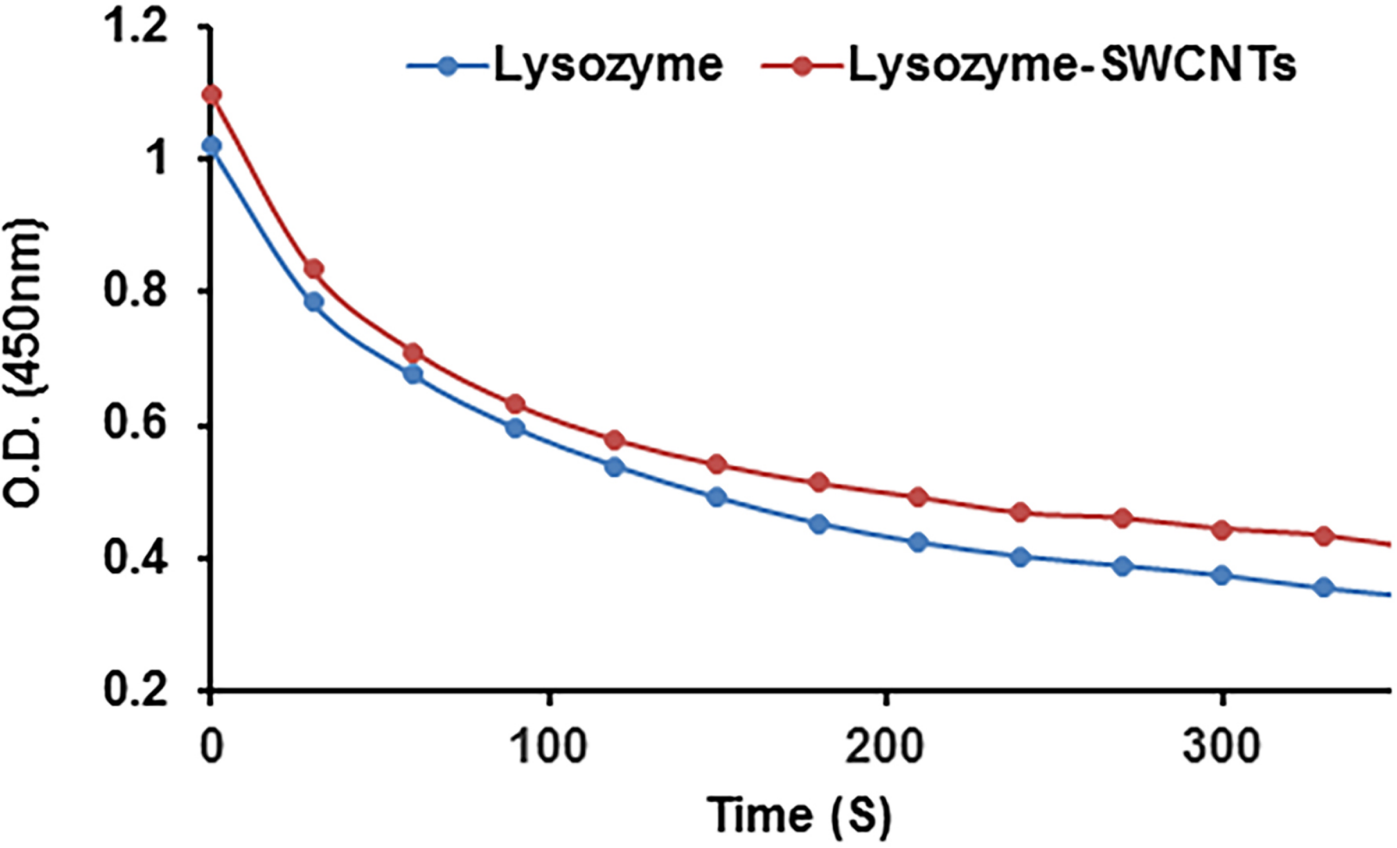
The kinetics of bacterial cell lysis was monitored as represented by a plot of O.D. at 450 nm vs. time (Lysozyme (blue) and lysozyme-SWCNTs (red)).

Using Imoto and Yagishita method [41] we showed that the lysozyme conjugated onto the SWCNTs retained its lytic activity, Fig 4. We found that with time the absorbance decreases more sharply in the case of the lysozyme-SWCNTs same as free lysozyme. The results showed that in the case of the lysozyme-SWCNTs the lysis of bacterial cells was slightly lower than the free lysozyme.

### Length-dependent separation of bio-conjugated SWCNTs using SDS-PAGE and visualization with silver staining

**Fig 5:**
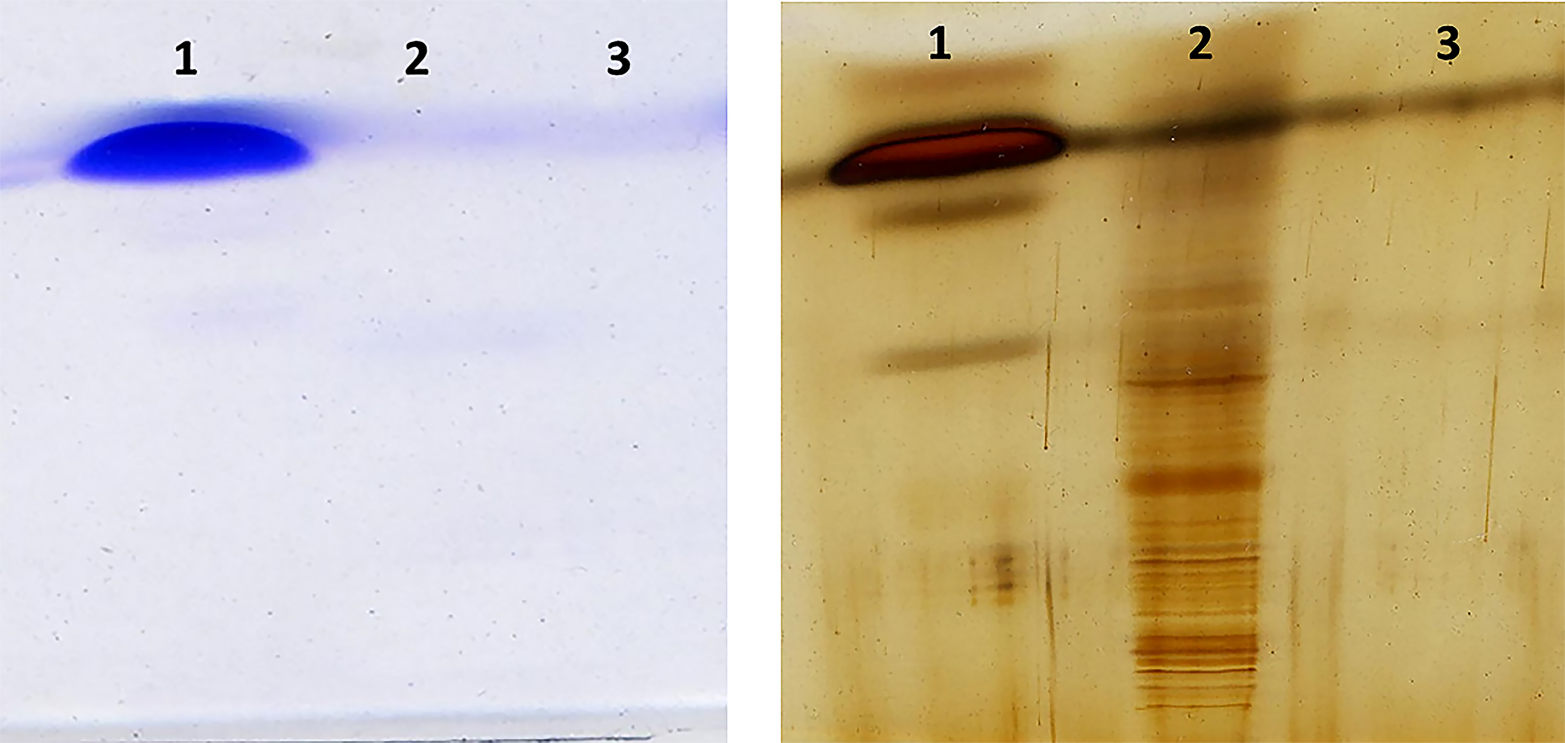
SDS-PAGE electrophoresis of lysozyme, SWCNTs, and conjugated lysozyme with coomassie blue staining (left), and silver staining (right). Lane 1 is lysozyme, lane 2 is conjugated lysozyme-SWCNT, and lane 3 is SWCNT.

Because of lysozyme ability to disperse CNTs, conjugation of lysozyme and CNTs are of particular interest [40]. Based on molecular modelling [42] and experimental studies, lysozyme has the potential to sort nanotubes based on diameter. In this study, we used the conjugation of lysozyme onto carboxyl functionalized carbon nanotubes as a tool to separate carbon nanotubes by length. Selective visualization of nanotube fragments in the acrylamide gel which is a challenge for a number of nano-tech researchers, was achieved using silver staining. In contrast to the coomassie blue staining, silver staining showed high resolution CNTs length-based separation of lysozyme-SWCNT fragments. Fig 5(left) shows coomassie blue staining and 5(right) shows silver staining of free lysozyme (lane 1), conjugated lysozyme-SWCNTs (lane 2), and SWCNTs (lane 3).

The coomassie dyes (R-250 and G-250) are anionic dyes that stoichiometrically bind to proteins through ionic interactions between dye sulfonic acid groups and positive protein amine groups as well as through Van der Waals attractions.

Coomassie blue staining did not show the lysozyme-SWCNTs because of amide bond formation between primary amines of protein and carboxyl groups of SWCNTs, thus there would not be any binding between coomassie blue and lysozyme. In contrast, sharp bands were visualized with silver staining. The sharpness of the bands may be due to the stability of the conjugation, precise proportion of lysozyme molecules based on nanotube lengths and silver staining sensitivity to proteins. Conjugated lysozyme-SWCNT fragments with different lengths had individual mobilities.

### Length measurements of lysozyme-SWCNT fragments for different sonication intervals using image analysis techniques

**Fig 6:**
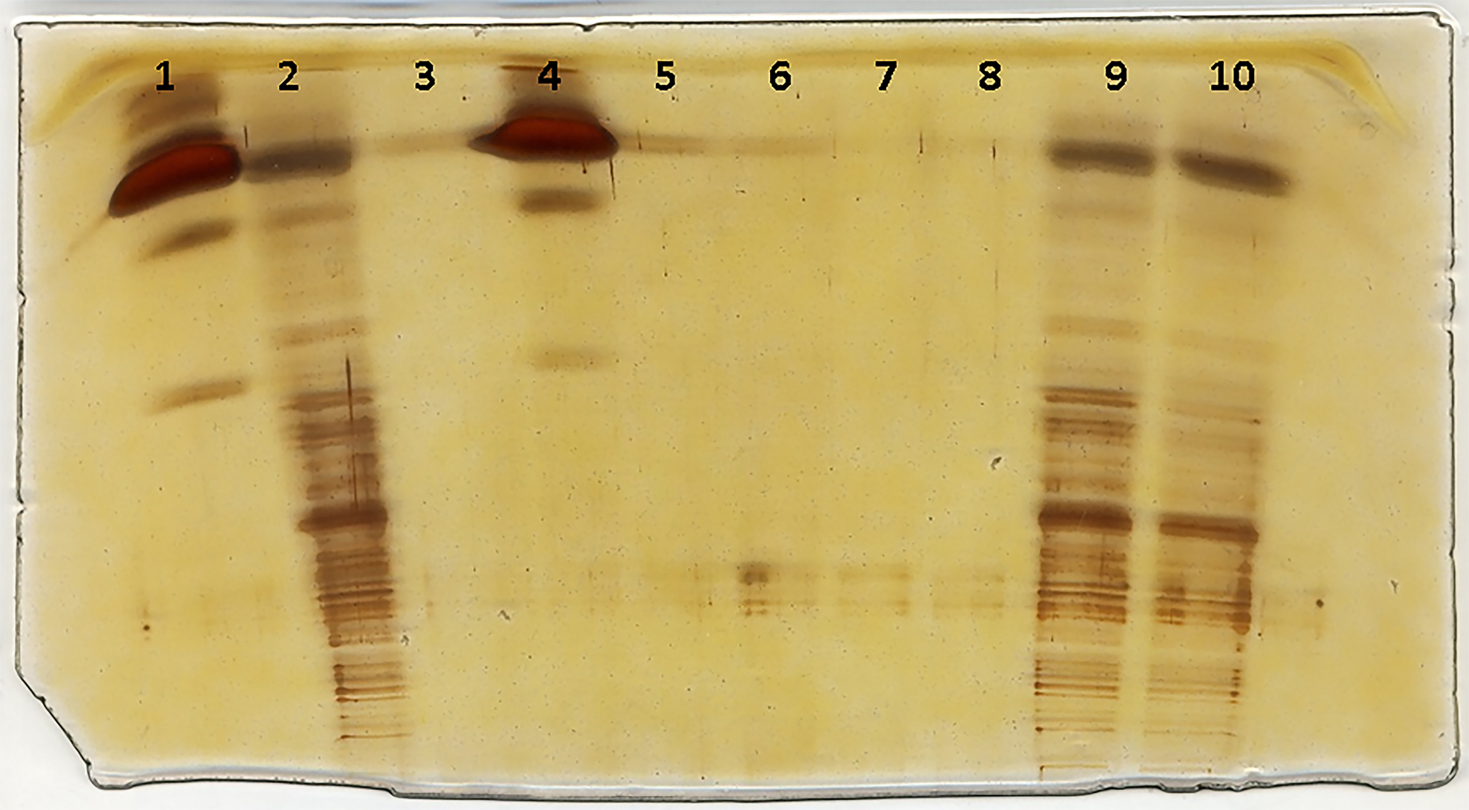
SDS-PAGE and silver staining of sonicated samples of lysozyme-SWCNTs, lanes 1 and 4 are free lysozyme, and lane 3 is SWCNTs, lane 2, 9, and 10 are sonicated conjugated lysozyme-SWCNT samples for 3, 7, and 10 min, respectively. It is clear the conjugated SWCNTs are separated based on their lengths.

Produced lysozyme-SWCNTs were sonicated for three periods (3, 7, and 10 minutes) followed by a SDS-PAGE. To visualize different degrees of nanotube migration into the gel, the bio-conjugated lysozyme-SWCNTs were silver-stained. The migration results are shown in Fig 6.

Lane 3 shows the SWCNTs with no apparent mobility and lanes 2, 9 and 10 show the migration of sonicated lysozyme-SWCNT samples for 3, 7, and 10 minutes. Lanes 1 and 4 show the free lysozyme.

The corresponding nanotube length of CNTs were calculated from the following theoretical formula [34],

L=d×exp([3πμη/(q(d)×e)−2 ln(2)+1]) (1)

where d = 89.0 ± 0.2 (nm) is the average diameter of each CNT and η = viscosity = 1.25 (Pa.s),. q(d) is calculated according to Usrey et al. and e is the electron charge [34].

**Fig 7:**
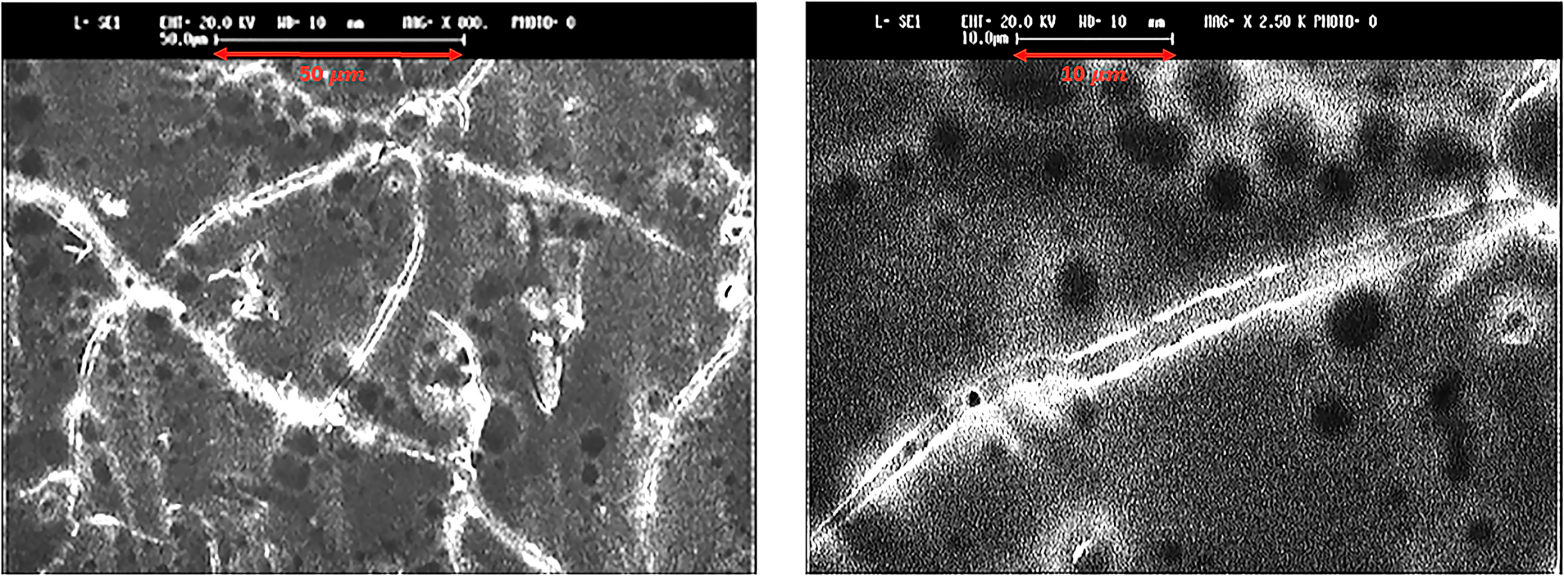
Scanning electron micrographs of conjugated lysozyme-SWCNTs.

Visual evaluation confirms that the average length of CNTs is between 45–65 microns (Fig 7).

In this study, two methods were used to calculate the length of CNTs. In the first method, a computer program was developed in MATLAB that subtracts the background, selects 3 lines on each lane of the gel and averages the signal (intensities of the bands) at each distance from the center of the wells. In the second method, ImageJ is used to calculate the same parameter using a narrow rectangle along each lane from the well to the bottom of the gel. These methods generated similar results (as shown in Fig 7) that are in concordance with the visual evaluations.

**Fig 8:**
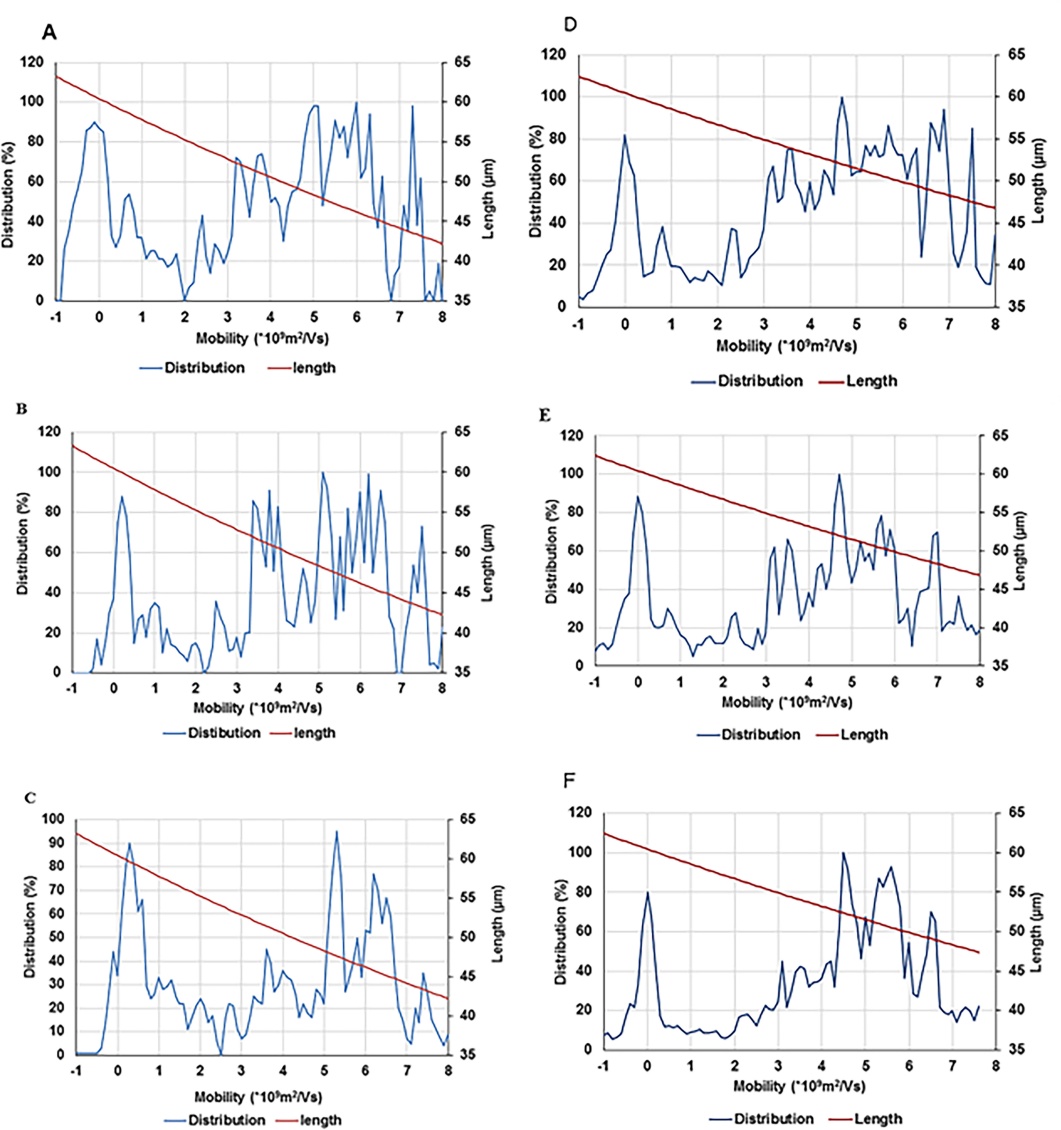
Distribution and the lengths of SWCNT fragments in two methods (MATLAB, 8A through 8C & ImageJ, 8D through 8F) after sonication time at 3 min (8A & 8D), 7 min (8B & 8E), and 10 min (8C & 8F). The length of CNTs are in concordance with two methods ranging between 43 to 63 microns when calculated using MATLAB and 47 to 63 microns when ImageJ is used.

After an analysis of gel images, experimental data were obtained in the form of mobility distribution (number of nanotubes as a function of mobility). As Fig 8 shows, SWCNTs of various lengths are present in the population for each experimental electrophoretic mobility value.

Colour was considered as a feature used in image database retrieval. Two methods were used to measure the distribution of the signal on images acquired from gels; in the first method, ImageJ were used to convert the coloured image to an 8 bits gray scale image and then a background subtraction algorithm was used to improve the signal to noise ratio. Then, the intensity of the bands at each distance from the wells was extracted using plot profile of a very narrow box. The second method uses an algorithm for colour matching developed in MATLAB. Both methods show comparable results.

## Discussion

Nanotubes may be produced in a wide range of length, diameter, and structure. Much research has focused on accurately measuring the structure-based purification of nanotubes because some of their properties are dependent on these structural parameters. Therefore, length-based separation will be an important tool for advancing nanotube science. In CNTs synthesis processes, accurate measurement of nanotubes length is important to understand nanotube growth and cutting processes [43]. Franklin and his colleagues provided the first experimental evidence of the effects of contact length in nanotube transistors by fabricating sets of devices with different nanotube lengths [10]. Moreover, the length-based separation of CNTs is considered a key step enabling their applications in biologically relevant settings such as drug delivery.

Our results from XRD patterns (Fig 1), FTIR (Fig 2), and SEM micrographs (Fig 3A and 3B) showed that lysozyme disposed onto the SWCNTs surface completely during conjugation. The presence of covalently attached proteins gives rise to a functionalization charge and an intrinsic positive charge, respectively, which together comprise the net charge on any given individual nanotube or bundle in solution. High precision separation is carried out because the amount of conjugated lysozyme on SWCNT fragments is proportional to their lengths. In other words, net charge of fragments is directly proportional to the amount of conjugated lysozyme. It forces SWCNT fragments with different length to move with different speed in an electrophoretic field. The mobility and velocity of charged CNT fragments depend directly on the electrical field (E, volts/cm) and net charge on the CNT fragments (q) but, inversely on the friction of the molecules.

$$V=\;\frac{Eq}{f}$$ (2)

where f = frictional coefficient of the mass and shape of the fragment and V = velocity/mobility of the fragment [42]. Smaller CNTs with higher mobility can pass through the gel grid more easily when compared with the larger nanotubes. In other words, the gel acts like a sieve and retains the larger nanotubes while allowing the smaller ones pass through. Therefore, the frictional coefficient is related to how easily a SWCNT fragment passes through the pores of the gel. Indeed, length will be the major determinant of the mobility of SWCNTs in a gel matrix. Substituting length for the frictional coefficient results in: mobility = (voltage)(charge)/(length). In other words, the mobility of a SWCNT fragment during gel electrophoresis is primarily a function of its charge/length ratio.

**Fig 9:**
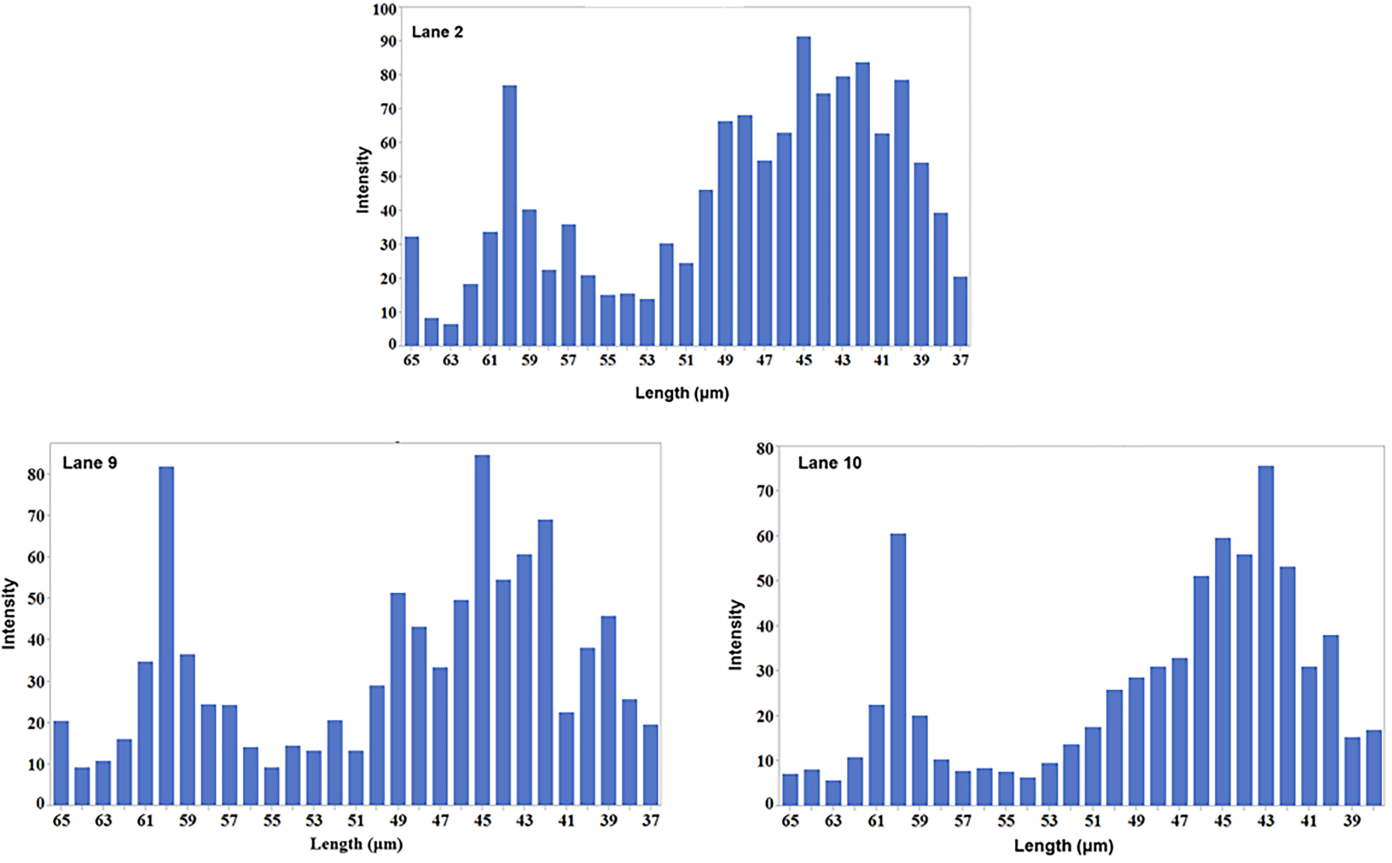
Length distribution of the conjugated SWCNTs. **The intensity of the CNTs at each lane is plotted versus length of CNTs calculated from Eq (1)**.

The length distribution of the conjugated SWCNTs can be represented by the distribution of the calculated lengths from the Usrey’s equation (Eq (1)) versus the intensity of the bands of the lanes (Fig 9A, 9B, and 9C).

The intensity can be viewed as a measure of the number of CNTs with a specific length on the gel; the higher the intensity, the more CNTs with that specific length is at that lane. It is obvious that the main distribution of the lengths of CNTs are between 38–50 microns. There is another peak at 60 microns which is for the CNTs in the well which had not yet departed the well.

One problem in CNT-based nanobiotechnology, nanomedicine, and CNT-based molecular electronics is the lack of length uniformity in mass production of CNTs. In these applications, it is important to separate CNTs based on their length and conductivity. For the first time, we have demonstrated a method that combines bio-conjugation, SDS-PAGE, and silver staining in order to perform a lengthwise separation of CNTs. This method is a valuable quality control tool for the manufacture of carbon nanotubes of specific lengths in bulk quantities.

Since 1998, there have been more than 200 papers on the separation of SWNTs based on their conductivity, diameter, handedness, and length in open literature, Table 1.

**Table 1:** Length-based separation methods for SWCNTs.

CNT	Method	Reference
SWCNT	Electrophoresis	[31]
SWCNT	Electrophoresis	[44]
SWCNT	Electrophoresis	[32]
SWCNT	Density gradient ultracentrifugation (DGU).	[45]
SWCNT	Chromatography	[46–50]
SWCNT	Chromatography	[51–53]
SWCNT	Chromatography	[25, 26], [54, 55]
SWCNT	Chromatography	[56]
SWCNT	Chromatography	[57]
SWCNT	Chromatography	[24]
SWCNT	Chromatography	[58]
SWCNT	Chromatography	[59]
SWCNT	Selective solubilization	[60]

Several chromatographic methods have been reported for length-based separation of CNT’s by Georg Duesberg and his colleagues[25, 54, 61]using Size-exclusion chromatography (SEC), Andrew Rinzler and his team[62] using high performance liquid chromatography (HPLC) and Fotios Papadimitrakopolous’s group[63] using gel permeation chromatography, to name a few. Using chromatography, it is difficult to predict the precise time that any given particle will exit the column because of the stochastic nature of the particle-pore interactions. Thus, chromatographic separation processes are not known as capable methods for extracting carbon nanotubes of specific lengths. Beside centrifugation[64], length-based separation of carbon nanotubes by their electrical properties is more commonly performed using capillary electrophoresis(CE) and agarose gel electrophoresis[31, 32]. However, there exist no electrophoretic methods for precise and rapid length-based separation of CNTs.

The aforementioned methods which have poor precision and scalability, used the UV/vis spectroscopic, AFM, and Raman Spectroscopy. These techniques are complex and expensive while our unconventional method which addresses an important and challenging problem in materials science is very precise, versatile, rapid and inexpensive for length-based separation of SWNTs.

Silver staining with an excellent sensitivity was used to visualize lysozyme-SWCNT fragments with high-resolution [65]. In this research, our method combined bio-conjugation of nanotubes with a high precision staining technique with high sensitivity (in the low nanogram range) whilst using very simple and inexpensive equipment and chemicals. Conjugation with a biomolecule led to the length-based separation and silver staining helped to visualize the yield SWCNTs fragments.

It is worth mentioning that other proteins could also be utilized for this purpose. As illustrated in this research, our concept of sorting and length-based separation of CNTs using bio-conjugation, SDS-PAGE, and silver staining is validated by the theoretical model of Usrey et al. [34]. Bio-conjugates include a large group of proteins such as peptides and other biomolecules. This novel method is successfully validated for the first time.

Consequently, this method with high efficiency, which provides a better estimation of the nanotube length, can be a valuable quality-control tool when bulk quantities of pure CNTs with identical lengths are desirable.

## Conclusions

We have demonstrated a combination of bio-conjugation, SDS-PAGE, and silver staining to length-based separate CNTs and to measure the lengths of the resulted nanotubes using electrophoretic mobility values in an acrylamide gel.

While a few researchers have focused on the length-based separation of CNTs using gel permeation chromatography column and an inhomogeneous magnetic field, none to date have reported the use of conjugation with a biomolecule to length-based separate and silver stain to visualize carbon nanotube fragments. Although previous studies reported the use of detergents such as Triton X-405 [36] and SDS [43] for agarose gel electrophoresis, the current study uses an high precision, inexpensive, rapid and simple SDS-PAGE and silver staining procedure, for high precision separation and high-resolution visualization. In addition, this method obviates the need for any further testing, such as Raman spectroscopy, because of clear and easy observation of SWCNT fragments with naked eye.
